# The effect of social exclusion on aggressive behavior among Chinese college students: the mediating role of relative deprivation and the moderating role of upward social comparison

**DOI:** 10.3389/fpubh.2025.1632073

**Published:** 2025-09-23

**Authors:** Xiaofang Yu, Gensen Xiao, Yanzhen Zhang

**Affiliations:** ^1^Jiangxi Normal University, Nanchang, China; ^2^UCI Donald Bren School of Information and Computer Sciences, Department of Computer Science, University of California, Irvine, Irvine, CA, United States

**Keywords:** social exclusion, aggressive behavior, relative deprivation, USC, mediating role

## Abstract

**Background:**

The current study examined the effect of social exclusion on aggressive behavior, how relative deprivation might mediate this effect, and how upward social comparison (USC) might moderate the indirect pathway.

**Methods:**

One thousand seven hundred and sixty-six college students were investigated, with an average age of 19.53 (SD = 1.09) years. Participants completed questionnaires regarding social exclusion, aggressive behavior, relative deprivation, and USC. The data was analyzed using regression-based moderated mediation modeling. PROCESS Models 4 and 7 macros for SPSS were used to test the mediation and moderated mediation models with 5,000 random sample bootstrapping confidence intervals (CIs).

**Results:**

The findings revealed a significant positive association between social exclusion and aggressive behavior among Chinese college students (*r* = 0.362, *p* < 0.001). Relative deprivation played a partial mediating role between social exclusion and aggressive behavior (indirect effect = 0.045, 95%CI [0.028, 0.062]). The association between social exclusion and aggressive behavior was moderated by USC. For college students with low USC, the effect of moderated mediation (effect = 0.035, 95%CI [0.022, 0.050]). For college students with high USC, the effect of moderated mediation was 0.057 (95%CI [0.034, 0.081]). The link between social exclusion and relative deprivation was stronger for college students with high levels of upward social comparison than for college students with low levels of upward social comparison (β = 0.405, *t* = 11.976, *p* < 0.001 vs. β = 0.251, *t* = 8.182, *p* < 0.001).

**Conclusion:**

Relative deprivation could be a mechanism by which social exclusion was linked with aggressive behavior and USC enhanced the effect of relative deprivation. This study was important in investigating how social exclusion was related to aggressive behavior among Chinese college students which provided meaningful implications for reducing aggressive behavior. Thus, this study explored “how” and “when” social exclusion might enhance aggressive behavior among Chinese college students. The results suggested that relative deprivation and USC might be prime targets for prevention and intervention programs of aggressive behavior among Chinese college students.

## 1 Introduction

Aggressive behavior, which is intended to be associated with harm to other people, is an important indicator for measuring individual social adaptation (1). A survey shows that aggression has become the third leading cause of death for individuals aged 10 to 24 and is related to medical losses exceeding $21 billion annually (2). 10.7% of individuals have engaged in aggressive behavior toward others, and 3.6% are both aggressors and victims (3). The continuous occurrence of campus violence incidents in recent years has aroused high attention to aggressive behavior (4, 5). College students’ aggressive behavior is also increasing. For example, research shows that 88.8% of Chinese college students displayed various aggressive behaviors (6). Aggressive behavior can seriously threaten individuals’ physical and mental health as well as social behavior, and increase crime rates (7). Therefore, due to the high incidence of college students’ aggressive behavior and the adverse consequences that aggressive behavior brings, it is essential to examine the influencing factors of college students’ aggressive behavior, which facilitates the advancement of aggressive behavior prevention.

Empirical studies indicate a positive correlation between social exclusion and aggressive behavior. However, there are few studies to examine the potential mediating and moderating mechanisms in this association. This study explored how social exclusion exacerbated relative deprivation, which in turn increased aggressive behavior. Further, this study examined whether social exclusion and USC interacted in a manner such that USC enhanced the effect of social exclusion on relative deprivation.

### 1.1 Social exclusion and aggressive behavior

Social exclusion is linked with a decrease in an individual’s self-esteem, invokes negative emotions, and increases the likelihood of externalizing problematic behaviors (8, 9). The cognitive linking model also shows that setbacks (e.g., social exclusion) is linked with individuals’ negative emotions that individuals repeatedly attend to, and when they are in a similar situation again, negative stimuli make individuals pay more attention to the negative information, thereby activating their tendency to attack and increasing the likelihood of aggressive behavior (10). According to the cognitive linking model, social exclusion, as a negative stimulus, may induce negative emotions and hostile cognition in individuals, leading to aggressive behavior. Empirical studies have also found that individuals who are excluded are likely to choose less attractive foods for their interacting peers (11), make louder noises (12), and allocate more spicy sauce (13). Moreover, social exclusion may also increase attacks on unrelated individuals. Empirical studies also indicate a positive correlation between social exclusion and aggressive behavior (14, 15).

### 1.2 The mediation effect of relative deprivation

Relative deprivation refers to subjective cognitive and negative emotional experience when an individual or group perceives their position and situation as inferior compared to other individuals or groups, and subsequently experiences the deprivation of their basic rights (16). The frustration attack theory suggests that relative deprivation can induce individual deviant behavior (17). The classic theory of relative deprivation suggests that individuals primarily assess their situation and status by comparing themselves with others, and vulnerable individuals in the group may feel deprived of their basic rights by individuals in the group, which is related to severe damage to their physical and mental development (18). High relative deprivation is linked with individual psychological development and is linked with aggression (19, 20). When individuals perceive discrimination and disadvantage, they feel social injustice and develop a sense of relative deprivation, which induces individuals to attack others. Research reveals a strong positive correlation between violent conduct and relative deprivation (21, 22). Negative interpersonal conflicts in reality can deprive individuals of the opportunity to obtain social connections, inducing them into a relatively deprived state (23). Due to the exclusion itself being a product of power imbalance, there exists opposition between the advantaged and the disadvantaged, and when individuals are excluded, they are highly susceptible to experiencing relative deprivation through social comparison processes (24, 25). Research has found that long-term exclusion can lead individuals to perceive more discrimination and negative experiences when they compare themselves to others in society, making them more likely to experience relative deprivation (26). Long-term social exclusion is related to less confidence and more insecurity (27), reports more discrimination and adverse situations (28), and experiences a sense of relative deprivation. This study hypothesized that relative deprivation acted as a mediator between social exclusion and aggressive behavior.

### 1.3 The moderating role of USC

USC is more likely to pose a threat to individuals, which may induce negative emotions and create frustration in individuals (29). Social comparison theory suggests that comparison is the process by which individuals establish their self-worth by comparing themselves with others (30). According to the contrast effect of USC in the process of upward comparison with social comparison objects, when individuals feel that they cannot reach the level of the social comparison object in the future, the level of their self-evaluation, self-esteem, and self-worth moves away from the social comparison goal (31). Individuals lower the level of their self-evaluation, self-esteem, and self-worth when facing upward comparison information and feel frustrated, distressed, and disappointed (29, 32). Feinstein et al. believe that individuals have a universal upward drive, which drives them to strive for positive self-evaluation and acknowledge their abilities (33). When individuals compare themselves with others, they have feelings of jealousy (when they are lower than the other person) (34, 35). In the digital media era, the anonymity of the internet and the tendency of social media to showcase the best aspects of life exacerbate upward comparisons among individuals (36). In a collectivistic context, individuals are more likely to engage in upward comparison with ingroup members, and falling short of the group average may elicit a sense of guilt for “pulling the group down.” Therefore, when individuals are surpassed by others, they are likely to generate dissatisfaction and have negative experiences (37, 38). That is to say, when compared to individuals who are more capable than oneself, the perception of one’s abilities is hindered, leading to feelings of frustration and relative deprivation (39). Therefore, there is a positive correlation between USC and relative deprivation. The higher the degree to which an individual engages in USC, the more severe the relative deprivation (40, 41). The risk-enhancing model shows that one risk factor enhances the effect of another risk factor, and the effect of a single risk is relatively limited, but when some risks accumulate, the impact is no longer simply the sum of two risks, but rather brings greater adaptation difficulties (42–44). According to the risk-enhancing model, for college students with low levels of USC, the effect of social exclusion on relative deprivation was stronger than for college students with high levels of USC. Therefore, USC may moderate the relationship between social exclusion and relative deprivation.

### 1.4 The present study

**Figure 1:**
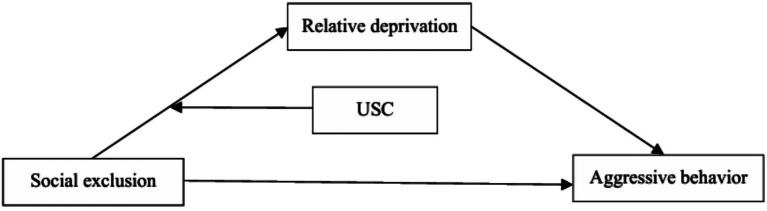
Conceptual model.

Previous research had not explored the relationship among social exclusion, relative deprivation, social comparison, and aggressive behavior. The current study addressed this gap. This study explored “how” and “when” social exclusion might enhance aggressive behavior among Chinese college students. To summarize, the current study had two aims. First, the study evaluated whether relative deprivation mediated the relationship between social exclusion and aggressive behavior. Second, this study examined whether USC moderated the associations between social exclusion and relative deprivation (Figure 1). This study put forward two hypotheses:

*Hypothesis 1*. The effect of social exclusion on aggressive behavior would be mediated by relative deprivation.

*Hypothesis 2*. USC would moderate the relationship between social exclusion and relative deprivation. USC would moderate the indirect relations between social exclusion and social exclusion via relative deprivation. The effect of social exclusion on relative deprivation would be enhanced by USC.

## 2 Methods

### 2.1 Participants

In order to ensure a diverse and inclusive sample, this study selected participants from universities in eastern, western, southern, northern, and central China, respectively. Before data collection, participants’ consent was acquired, and participants completed the survey voluntarily with no compensation in the study. The mean completion time that participants completed questionnaires was 6.45 min (SD = 0.89). The upper and lower threshold was 3.78 min and 9.12 min. The criteria for unqualified participants were less than 3.72 min and more than 9.12 min to complete questionnaires, and having missing data and regularity of answers, such as the same score or a regular pattern of scores (i.e., the same option being selected in each item or answer in the pattern of 5, 4, 3, 2, 1).

After excluding unqualified participants, 1,766 (Mage = 19.53, SDage = 1.09) valid questionnaires were finally collected, with an effective response rate of 97.53% from 1,811 primary questionnaires. The mean age ranges from 18 to 23 years. 56.60% of participants were females. 29.8% of participants were rural, and 70.2% of participants were urban. 10.5% of participants ‘majors were philosophy, 10.7% of participants’ majors were medicine, 26.9% of participants’ majors were science, 14.6% of participants’ majors were humanities, 27.8% of participants’ majors were engineering, and 9.5% of participants’ majors were arts. 24.3% were freshmen, 26.4% were sophomores, 25.7% were juniors, and 23.6% were seniors. 19.3% were from eastern, 19% were from western, 20.4% were from southern, 20.2% were from northern, and 21.1% were from central China. 31.7% of participants’ families had a monthly income below 3,000, 36.2% of participants’ families had a monthly income between 3,000 and 6,000, 16.4% of participants ‘families had a monthly income between 6,000 and 9,000, and 15.7% of participants had a monthly income over 9,000. The sample size estimation was performed using G * Power 3.1.9.4 software in advance (45) to examine a small effect (*r* = 0.10), requiring 1,289 participants and providing 95% statistical power. Therefore, the sample size of this study could provide at least 95% statistical power.

### 2.2 Aggression questionnaire

Aggression was measured by the Inventory of Aggression, which was originally developed by Buss and Perry (46). The Chinese version was revised by Lv et al. (47). This scale consists of 22 items (e.g., “Given enough provocation, I may hit another person”) and includes four dimensions: hostility, physical aggression, impulsivity, and anger proneness. Each item was rated on a five-point scale (1 = extremely uncharacteristic of me to 5 = extremely characteristic of me). Reverse items were reverse-coded. The average score of 22 items was calculated, with higher scores reflecting more severe Aggression. In the present study, Cronbach’s alpha was 0.89. Furthermore, confirmatory factor analysis (CFA) suggested that the four-factor model fitted the data well: TLI = 0.94, CFI = 0.96, RMSEA = 0.08, SRMR = 0.05.

### 2.3 Social exclusion questionnaire

The Chinese version of the social exclusion Questionnaire (48) was used to measure social exclusion. Participants rated 19 items on a five-point scale ranging from 1 (never) to 5 (often), including direct exclusion (e.g., “Everyone intentionally or unintentionally avoids me when joking or playing around with each other”) and indirect exclusion (e.g., When I feel lost, I cannot receive advice or comfort from others). The average score of the 19 items was calculated, with higher scores reflecting greater social exclusion. In the present study, Cronbach’s alpha for the scale was 0.90.

### 2.4 Relative deprivation questionnaire

The Chinese version of the relative deprivation Questionnaire (49) was used to measure social exclusion. Participants rated four items (e.g., I always feel that someone else has taken what should have belonged to me) on a six-point scale ranging from 1 (strongly disagree) to 6 (strongly agree). The average score of the four items was calculated, with higher scores reflecting greater relative deprivation. In the present study, Cronbach’s alpha for the scale was 0.90.

### 2.5 USC scale

USC was measured by the USC Scale, which was originally developed by Gibbons and Buunk (50) and revised the Chinese version by Bai et al. (51). This scale consists of six items (e.g., “I often like to compare myself with those who are doing better than me”). Each item was rated on a five-point scale (1 = strongly disagree to 5 = strongly agree). The average score of six items was calculated, with higher scores reflecting great USC. In the present study, Cronbach’s alpha for the scale was 0.8.

### 2.6 Statistical analyses

**Figure 2:**
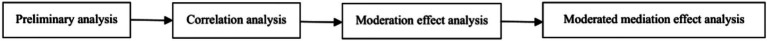
Research process flowchart.

Research process flowchart was showed in Figure 2. Preliminary analyses of univariate descriptive statistics (e.g., mean, standard deviation, reliability) and bi-variate correlations were calculated using SPSS26. PROCESS Models 4 and 7 macros for SPSS were used to test the mediation and moderated mediation models with 5,000 random sample bootstrapping confidence intervals (CIs) using SPSS26. In the meantime, the demographic variables (gender, grade, origin, and only-child or not) and were controlled. The product of the demographic variables (gender, grade, origin and only-child or not, major, age, and monthly family income) and upward social comparison were also controlled. An effect is regarded as significant if the CIs do not include zero. Gender, grade, origin, and only-child or not were controlled in the analyses. TRIPOD guidelines were followed for reporting a predictive model.

## 3 Results

### 3.1 Preliminary analyses

The means and Pearson correlations among the study variables are presented in Table 1. Social exclusion was positively correlated with relative deprivation (*r* = 0.394, *p* < 0.001), USC (*r* = 0.186, *p* < 0.001), and aggressive behavior (*r* = 0.362, *p* < 0.001). Relative deprivation was positively correlated with USC (*r* = 0.288, *p* < 0.001) and aggressive behavior (*r* = 0.318, *p* < 0.001). Additionally, USC was positively correlated with aggressive behavior (*r* = 0.130, *p* < 0.001).

**Table 1:** Means, standard deviations, and correlations of the main study variables.

Variables	*M*	*SD*	1	2	3	4
1. Social exclusion	1.860	0.818	–			
2. Relative deprivation	3.151	0.909	0.394∗∗∗	–		
3. USC	3.242	0.467	0.186∗∗∗	0.288∗∗∗	–	
4. Aggressive behavior	3.190	0.861	0.362∗∗∗	0.318∗∗∗	0.130∗∗∗	–

USC, upward social comparison, ^∗∗∗^*p* < 0.001.

### 3.2 Evaluating the mediating role of relative deprivation

As Table 2 Equation 1 (aggressive behavior) showed, social exclusion was positively related to aggressive behavior (β = 0.260, *t* = 12.080, *p* < 0.001, 95%CI [0.213, 0.304]). In hypothesis 1, this study anticipated that the association between social exclusion and aggressive behavior was mediated by relative deprivation. Model 4 of Hayes’ SPSS macro-PROCESS was used to test this hypothesis. Table 2 shows the results of the regression analysis conducted to test mediation. According to Equation 2 (relative deprivation) and Equation 3 (aggressive behavior), social exclusion was significant positively related to relative deprivation (β = 0.322, *t* = 14.629, *p* < 0.001, 95%CI [0.279, 0.365]) and significant positively related to aggressive behavior (β = 0.215, *t* = 9.522, *p* < 0.001, 95%CI [0.171, 0.260]). Relative deprivation was positively associated with aggressive behavior (β = 0.140, *t* = 5.988, *p* < 0.001, 95%CI [0.131, 0.222]). The indirect effect of mediation was significant (indirect effect = 0.045, 95%CI [0.028, 0.062]). Thus, hypothesis 1 was supported, and relative deprivation partially mediated the relationship between social exclusion and aggressive behavior.

**Table 2:** Test of moderated mediation between social exclusion and aggressive behavior.

Variables	Equation 1 (AB)			Equation 2 (RD)			Equation 3 (AB)			Equation 4 (RD)		
	β	95% *CI*	*t*	β	95% *CI*	*t*	β	95% *CI*	*t*	β	95% *CI*	*t*
Grade	0.061	0.021, 0.098	3.071^***^	0.074	0.034, 0.113	3.647^***^	0.051	0.012, 0.089	2.567^*^	0.073	0.033, 0.112	3.597
Gender	0.141	0.049, 0.234	3.089^***^	0.057	−0.034, 0.148	1.224	0.133	0.044, 0.221	2.942^**^	0.064	0.038, 0.208	1.384
Origin	−0.068	−0.165, 0.033	−1.370	−0.048	−0.147, −0.051	−0.958	−0.061	−0.157, 0.035	−1.246	−0.049	−0.240, −0.008	−0.972
OCN	−0.106	−0.213, 0.003	−1.822	−0.128	−0.244, −0.012	−2.162^*^	−0.088	−0.249, −0.016	−1.527^*^	−0.124	−0.240, −0.008	−2.099
Major	0.043	0.014, 0.072	2.977^**^	−0.072	−0.101, −0.044	−4.916^***^	0.053	0.025, 0.081	3.689	−0.074	−0.102, −0.045	−5.011
Age	0.159	0.103, 0.215	6.101^***^	0.049	−0.004, 0.101	1.821	0.152	0.102, 0.203	5.894	0.048	−0.004, 0.100	1.804
MFI	0.201	0.152, 0.249	8.281	0.087	0.038, 0.135	3.510	0.188	0.141, 0.236	7.831	0.085	0.037, 0.133	3.438
Grade× SE	−0.025	−0.064, 0.014	−1.247	0.016	−0.024, 0.056	0.782	−0.027	−0.065, 0.012	−1.372	0.009	−0.032, 0.506	0.438
Gender× SE	−0.098	−0.193, −0.004	−2.100	0.009	−0.084, 0.102	0.185	−0.099	−0.189, −0.009	−2.148	−0.006	−0.010, 0.088	−0.122
Origin× SE	0.005	−0.079, 0.089	0.119	0.047	−0.045, 0.138	0.318	−0.001	0.038, 0.135	−0.023	0.050	−0.048, 0.147	0.994
OCN × SE	0.184	0.095, 0.274	3.759	0.081	−0.017, 0.180	1.625	0.173	0.078, 0.268	3.560	0.054	−0.060, 0.167	0.930
Major× SE	0.004	−0.022, 0.030	0.267	0.017	−0.010, 0.044	1.216	0.001	0.038, 0.135	0.095	0.012	−0.018, 0.041	0.778
Age× SE	0.021	−0.024, 0.071	0.904	−0.062	−0.109, −0.015	−2.594^**^	0.030	−0.016, 0.075	1.295	−0.060	−0.108, −0.012	−2.466
MFI × SE	−0.053	−0.104, −0.004	−2.231	0.009	−0.039, 0.056	0.354	−0.054	−0.100, −0.008	−2.304	0.027	−0.022, 0.076	1.076
SE	0.260	0.213, 0.304	12.080^***^	0.322	0.279, 0.365	14.629^***^	0.215	0.171, 0.260	9.522^***^	0.328	0.285, 0.371	14.880^***^
USC										0.055	0.164, 0.249	0.374
USC × SE										0.077	0.030, 0.124	3.224^***^
RD							0.140	0.131, 0.222	5.988^***^			
*R* ^2^	0.284			0.253			0.298			0.258		
*F*	45.603^***^			39.054^***^			45.857^***^			35.276^***^		

^∗^*p* < 0.05, ^∗∗^*p* < 0.01, ^∗∗∗^*p* < 0.001. OCN, only-child or not; MFI, monthly family income; SE, social exclusion; USC, upward social comparison; RD, relative deprivation; AB, aggressive behavior.

### 3.3 Moderated mediation effect analysis

**Figure 3:**
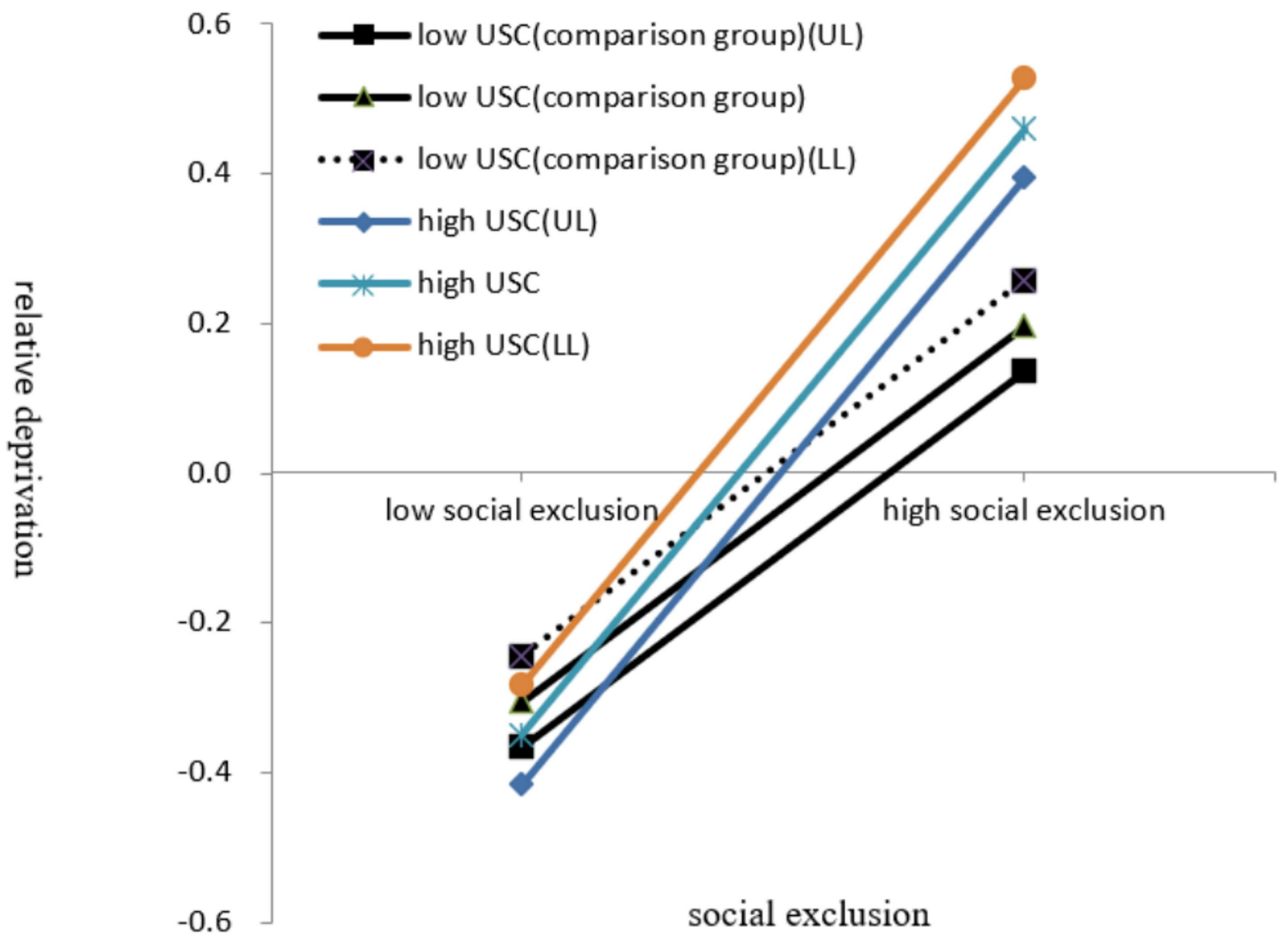
Interaction between social exclusion and USC on relative deprivation. UL, Upper limit of 95% confidence interval; LL, lower limit of 95%confidence interval.

The moderated mediation model was evaluated with Model 7 of the SPSS macro-PROCESS. The results are shown in Equation 4 of Table 2. The product of social exclusion and USC (the interaction term) was significantly associated with relative deprivation (β = 0.077, *t* = 3.224, *p* < 0.001, 95%CI [0.030, 0.124]), suggesting that USC could moderate the relationship between social exclusion and relative deprivation. Specifically, USC could moderate the first half of the indirect pathway. Hypothesis 2 was supported. For college students with low upward social comparison, the effect of moderated mediation was significant (effect = 0.057, 95%CI [0.034, 0.081]). For college students with high upward social comparison, the effect of moderated mediation was significant (effect = 0.035, 95%CI [0.022, 0.050]). For descriptive purposes, this study plotted and explored social exclusion against relative deprivation, separately for high USC and low USC (comparison group). The interaction effect was visually plotted in Figure 3 that included 95% confidence intervals and clearly labeled comparison groups (low USC). Simple slope tests showed that for college students with high USC, social exclusion was significantly associated with aggressive behavior (β = 0.405, *t* = 11.976, *p* < 0.001, 95% CI [0.339, 0.472]). As for college students with low USC, social exclusion was also significantly associated with aggressive behavior (β = 0.251, *t* = 8.182, *p* < 0.001, 95% CI [0.192, 0.313]). However, for college students with high levels of USC, the effect of social exclusion on relative deprivation was stronger than for college students with low levels of USC, demonstrating that USC acted as an enhancer in the relationship between social exclusion and relative deprivation.

## 4 Discussion

This study explored the effects of social exclusion on aggressive behavior among Chinese college students. Through a survey of 1,766 Chinese college students, the results suggested that social exclusion was significantly positively associated with aggressive behavior among Chinese college students. After verifying the direct link, this study constructed and evaluated a moderated mediation model to explore the mechanism of social exclusion on aggressive behavior. This study further found that relative deprivation played a partial mediating role between social exclusion and aggressive behavior among college students. USC moderated the relationship between social exclusion and relative deprivation. Therefore, this study had a clear grasp of how and when social exclusion was associated with aggressive behavior.

### 4.1 Social exclusion and aggressive behavior

The findings revealed that social exclusion was strongly associated with aggressive behavior among Chinese college students. It suggested that college students who suffered from more social exclusion were more prone to engage in aggressive behavior, which supports the cognitive linking model. The result was consistent with previous research (15, 16), which proposes that social exclusion was positively correlated with aggressive behavior. The higher the degree of social exclusion an individual experiences, the more likely they are to attack others. This may be due to Chinese college students experiencing negative emotions after being socially excluded, leading to increased stress, which increases stress and hinders the development of personal autonomy and effective conflict resolution skills, and inability to effectively protect themselves during conflicts. Meanwhile, socially excluded Chinese college students may learn social ways and strengths and may relieve stress and excrete emotions by excluding others. The general attack model suggests that individuals who are frequently socially excluded are more susceptible and more likely to make risky decisions (52). After experiencing the sense of compensation brought by excluding others, college students are highly likely to be trapped in a vicious cycle of social exclusion and aggression, making them more prone to engaging in aggressive behavior toward others. Individuals who are socially excluded are prone to higher susceptibility (52), are prone to impulsiveness, and underestimate the consequences, leading to the phenomenon of “violent desensitization” (6), which in turn leads to aggressive behavior. This shows that college students’ social exclusion should be a concern, and college students’ social exclusion should be reduced so as to reduce their aggressive behavior.

### 4.2 The mediation role of relative deprivation

Based on verifying the relationship between social exclusion and aggressive behavior among Chinese college students, this study also deeply explored the mediating effect of relative deprivation on social exclusion and aggressive behavior among Chinese college students. Our study showed that relative deprivation mediated the association between social exclusion and aggressive behavior among Chinese college students. That is, social exclusion affected aggressive behavior among Chinese college students through relative deprivation, which supported hypothesis 1. Therefore, social exclusion, and relative deprivation may be one of the underlying mechanisms for why some individuals are likely to have aggressive behavior. In the mediation process of the relationship between social exclusion and aggressive behavior, social exclusion had enhanced relative deprivation, consistent with previous studies (15, 16). In the mediation process of the relationship between relative deprivation and aggressive behavior, college students with higher relative deprivation are more likely to have aggressive behavior, consistent with previous studies (27). Social exclusion, as a threatening source, not only leads to the breakdown of social connections and the emergence of persistent multiple deprivation disadvantages (24), but also is related to social cognitive biases and engagement in more negative behaviors (53). In the process of social comparison, individuals who are excluded often feel inferior and prone to feelings of inferiority, and they perceive more relative deprivation (54–56), experience negative emotions such as anger (54), which can be projected externally (55) and trigger individuals’ extremist behavior, leading to violent behavior and attack other, which was similar with our findings. Therefore, relative deprivation among college students needs to be especially concerned about, as it is especially important for aggressive behavior prevention among college students.

### 4.3 The moderating role of USC

This study also found that USC had an enhancer effect on the relationship between social exclusion and relative deprivation, that is, when the individual’s USC was high, the effect of social exclusion on relative deprivation was significant; and when the individual’s USC was low, the effect of social exclusion on relative deprivation was also significant and was stronger. According to this moderation model, it might be concluded that for college students who had experienced more social exclusion compared to college students who had experienced less social exclusion, it better reflects the developmental disadvantage among college students with more USC. This may be due to the fact that college students who had less USC were concerned about their disadvantaged situation after being socially excluded, feeling that their disadvantaged situation could not be improved, leading to the generation of relative deprivation. However, when college students who had more USC were socially excluded, they tended to feel inferior (28) and were angrier and more dissatisfied compared to individuals who were better than them, resulting in stronger relative deprivation. These findings contribute to reducing USC among college students in the future. Therefore, it is critical to lessen college students’ social isolation and USC.

### 4.4 Implications

The study has theory and practice implications. The study revealed that college students who have experienced social exclusion are more likely to attack others and are more likely to experience relative deprivation. The demand threat time model (56) suggests that after experiencing long-term social exclusion, individuals enter a phase of withdrawal, incapable of overcoming environmental exclusionary behavior and unable to put in effort to meet basic needs, ultimately leading to an increasing sense of alienation from the outside world, feeling frustrated, depressed, and lacking in value. This study supported the demand threat time model. Individuals who have experienced social exclusion face more interpersonal pressure and poorer peer relationships than individuals who have not experienced social exclusion (57). College students who have experienced social exclusion find it difficult to receive support and understanding from their peers as they face negative life events, their sense of belonging to the group is difficult to satisfy, and they are more likely to experience negative emotions such as loneliness. Schools should teach college students interpersonal communication skills, and college students should actively learn how to get along harmoniously with others and form good peer relationships. When college students have negative emotions due to social exclusion, they should actively seek help from teachers or parents, and if necessary, seek psychological counseling to avoid using extreme methods such as aggression to vent their emotions.

According to our research results, college students who have high social exclusion and high USC were most likely to experience more relative deprivation, so attention should be paid to reducing both their social exclusion and their USC. In order to prevent socially excluded individuals from attacking others, more attention should be paid to their relative deprivation, and timely intervention and control should be conducted. Reducing the level of social exclusion among college students is more likely to lower the relative deprivation. Reducing the level of college students’ USC makes it easier for them to experience less social exclusion and lower their level of deprivation.

## 5 Limitations and future directions

It is necessary to acknowledge some of the study’s limitations. First, this study’s use of a cross-sectional design restricts the ability to conclude causality. Experimental and longitudinal approaches may be used in future research to assess causation in more detail. Second, response bias may have influenced the result of the study, like in some studies that use solely self-reported data for data collection. Future studies may try to collect data from multiple informants (e.g., parents, peers, or teachers) to further evaluate these findings. Third, the study’s participants are college students in China. In contrast to many college students in other cultures who may live in different rooms or houses, take different courses, and engage in different activities, college students in China have multiple classmates who live in the same room, attend the same class, and take many of the same courses. This may be reflected in several ways in how students perceive social exclusion and limit generalization.

## 6 Conclusion

In sum, this study took a crucial step in exploring how social exclusion may be related to aggressive behavior among Chinese college students. This study showed that relative deprivation played a partial mediating role between social exclusion and aggressive behavior. Social exclusion was not only directly and positively related to aggressive behavior but also indirectly affected aggressive behavior through the mediating effect of college students’ relative deprivation. Hypotheses 1 was supported. Moreover, USC played a moderating role in the effect of social exclusion on relative deprivation, and the relationship between social exclusion and relative deprivation became stronger for college students with high USC. Hypotheses 2 was supported. This study explored “how” and “when” social exclusion might enhance aggressive behavior among Chinese college students. The results suggested that relative deprivation and USC may be prime targets for prevention and intervention programs of aggressive behavior among Chinese college students.

## Data Availability

The original contributions presented in the study are included in the article/supplementary material, further inquiries can be directed to the corresponding author.
